# Oregano Essential Oil and Purple Garlic Powder Effects on Intestinal Health, Microbiota Indicators and Antimicrobial Resistance as Feed Additives in Weaning Piglets

**DOI:** 10.3390/ani14010111

**Published:** 2023-12-28

**Authors:** Daniel Serrano-Jara, Jorge Rivera-Gomis, José Antonio Tornel, María José Jordán, Cristina Martínez-Conesa, María José Cubero Pablo

**Affiliations:** 1 Department of Comparative Anatomy and Pathology, Veterinary Medicine Faculty, Regional Campus of International Excellence “Campus Mare Nostrum”, University of Murcia, Espinardo, 30100 Murcia, Spain; d.serrano@umh.es; 2 Scotland’s Rural College (SRUC), Centre for Epidemiology and Planetary Health, Inverness, Scotland IV2 5NA, UK; 3 Dalland Hybrid España, S.A., Fortuna, 30620 Murcia, Spain; jatornel@dhe.es; 4 Research Group on Rainfed Agriculture for Rural Development, Department of Rural Development, Oenology and Sustainable Agriculture, Murcia Institute of Agri-Food and Environmental Research (IMIDA), La Alberca de Las Torres, 30150 Murcia, Spain; mariaj.jordan@carm.es (M.J.J.); cristina.martinez4@carm.es (C.M.-C.); 5 Animal Health Department, Veterinary Medicine Faculty, Regional Campus of International Excellence “Campus Mare Nostrum”, University of Murcia, Espinardo, 30100 Murcia, Spain; mjcubero@um.es

**Keywords:** weaned piglets, oregano, garlic, intestinal health, ZnO, antimicrobial resistance

## Abstract

**Simple Summary:**

We studied the impact of the bioactive components degradation of oregano essential oil and purple garlic powder during storage in silos, their effect on the morphometry of the jejunum and ileum and the cecal microbiota as intestinal health indicators in the piglets during the post-weaning. We also monitored antimicrobial resistance in the commensal indicator *Escherichia coli*. Histological parameters and intestinal microbiota were measured in 140 piglets weaned at 21 days of age. Seven dietary treatments were used: a negative control group (basal diet), a positive control group with zinc oxide (3000 mg/kg of food), two groups with oregano essential oil at 0.4% and 1.2%, respectively, two groups with purple garlic powder 0.4% and 2%, respectively, and a group with oregano essential oil with 1.2% combined with purple garlic powder with 2%. Each group of piglets received the treatment for seven weeks, from weaning, before samples were taken. Antibiotic resistance profiles were studied in 81 *Escherichia coli* strains from the cecal content. A progressive loss of the bioactive components of oregano essential oil and purple garlic powder was observed during the 34 days of storage. With purple garlic powder at 2% and oregano essential oil at 1.2%, their combination showed results like zinc oxide and even superior results in terms of the histological parameters studied and the counts of *Escherichia coli* and *Lactobacillus*. We observed high levels of resistance to antimicrobials of all categories. In general, the high doses of the additives studied showed the best results, obtaining levels like or higher than those offered by zinc oxide.

**Abstract:**

Finding alternatives to zinc oxide is a pressing issue for the pig production sector. We studied the impact of the bioactive components degradation of oregano essential oil (OEO) and purple garlic powder (PGP) during storage in silos, their effect on the morphometry of the jejunum and ileum and the cecal microbiota as intestinal health indicators in piglets during the post-weaning period. We also monitored antimicrobial resistance in the commensal indicator *E. coli.* Histological parameters and intestinal microbiota were measured in 140 piglets weaned at 21 days of age. Seven dietary treatments were used: a negative control group (basal diet), a positive control group with ZnO (3000 mg/kg of food), two groups with OEO at 0.4% and 1.2%, respectively, two groups with PGP 0.4% and 2%, respectively, and a group with OEO with 1.2% combined with PGP with 2%. Each group of piglets received the treatment for seven weeks, from weaning, before samples were taken. Antibiotic resistance profiles were measured in 81 *E. coli* strains. On this occasion, only the control groups, ZnO, OEO 1.2%, PGP 2% and OEO 1.2% + PGP 2% were used, and the samples were obtained from the cecal content. A progressive loss of the bioactive components of OEO and PGP was observed during the 34 days of storage (*p* < 0.05). PGP 2%, OEO 1.2% and their combination showed results similar to ZnO (*p* > 0.05), or superior in the study of intestinal morphometry and the values of *E. coli* and *Lactobacillus.* All categories showed high levels of resistance. Only the strains isolated from the OEO 1.2% group did not show resistance to colistin and presented the lowest resistance values. In general, high doses of the additives studied showed the best results, obtaining levels like or higher than those offered by ZnO.

## 1. Introduction

The use of antibiotics in intensive livestock farming as prophylactics or as growth promoters has led to the induction of new antimicrobial resistance (AMR) that poses a serious problem for public health [1]. This same problem is found with the use of zinc oxide (ZnO), a product widely implanted in pig farming that is frequently used prophylactically to reduce the development of digestive pathologies after weaning and improve growth performance [2]. However, ZnO also presents a risk to public health given the environmental contamination it generates through manure [3] and the development of resistance to antibiotics and heavy metals [4,5]. In the European Union, this situation has led to an increase in the restrictions on the use of antibiotics and the prohibition of dietary supplementation with ZnO in therapeutic doses [6]. Finding nutritional alternatives that allow for the maintenance of the anatomical, physiological, microbiological and immune balance of the intestine is one of the main tasks of the sector.

In pigs, to ensure proper nutritional absorption, the epithelium of the small intestine is completely renewed every 2 or 3 days [7]. At birth, the intestine of piglets is in a very immature state compared to other systems such as the muscular or nervous system [8]. The interaction with nutrients from breast milk induces their development and maturation [8,9]. However, the early weaning commonly practiced in intensive production (21–28 days) does not allow optimal maturation of the intestine [10]. Between the fourth and seventh day after weaning, the height of the villi can be reduced by 35%, although it can be restored after fourteen days [11]. The length of the villi is compromised between the third and seventh day [12] and increasing the depth of the crypts can also be a problem [13,14]. This situation, together with the dietary, social, and environmental changes that occur [15], can lead to transient anorexia, inflammation and intestinal imbalance that frequently evolves into diarrhea [15,16,17].

To control the development of AMR, the European Medicines Agency classifies antibiotics into four categories—A (avoid), B (restrict), C (caution) and D (prudence)—depending on its possible consequences on public health, the development of resistance and the need and possibility of being used in veterinary medicine [18]. This classification is used in surveillance programs that seek to control the appearance and persistence of AMR in animals intended for human consumption. For this, commensal indicator bacteria that are found in healthy animals and acquire resistance more quickly than other pathogens are used: *E. coli, Salmonella* spp., *Campylobacter* spp. and *Enterococcus* spp. [19].

Antimicrobials can also negatively affect commensal bacteria in the gut microbiota [20]. Some of them, like *Lactobacillus* spp., are involved in digestion and energy harvesting processes, in addition to having anti-inflammatory effects. However, after weaning their number decreases with respect to bacteria such as *E. coli*, *Salmonella* spp. and *Campylobacter* spp., associated with the development of diarrhea [21].

Phytogenics are botanical derivatives that have sometimes been used as growth promoters and immunological stimulants [22,23]. Among the natural products that have historically been used for the prevention and cure of diseases, within the *Alliaceae* family we find garlic (*Allium sativum*) [24,25,26]. The purple garlic powder (PGP) contains phytochemicals with synergistic effects between them [27] including the following: ajoenes (E-ajoene, Z-ajoene), thiosulfinates (allicin), vinyldithiines (2-vinyl-(4H)-1,3-dithiine, 3-vinyl-(4H)-1,2-dithiine), sulfides (diallyl disulfide (DADS) and diallyl trisulfide (DATS). Garlic extracts and their phytochemicals have various biological activities including anti-inflammatory [28], anticancer, antioxidant [29], antimicrobial [30] and antifungal properties [31]. One of the most active compounds is allicin (allyl thiosulfinate) coming, after the breaking of the parenchyma, from the reaction between the alliinase enzyme and the alliin. Its pharmacological effect is attributed to its antioxidant activity, as well as to its interaction with proteins that contain thiols. It has been previously reported that the quality of garlic, regarding the allicin content, varies depending on the variety, region and growth environment [32]. In Spain, the native ecotype named “Purple from Las Pedroñeras” is an important source of thiosulfinate and allicin [33]. Different researchers have pointed out the effectiveness of the inclusion of purple garlic powder (PGP), as a food additive, in the diets of broilers and pigs.

Oregano (*Origanum vulgare* L.) is an aromatic plant widely distributed in Asia and the Mediterranean area [34]. Oregano essential oil (OEO) is a volatile extract from this plant, which has been described for the prevention of intestinal architecture distortion and as an additive capable of increasing the height of the villi [35]. OEO comprises more than twenty major components, including a large percentage of phenolic compounds with antioxidant properties [36] that have been described both in vitro and in vivo [37]. They also exhibit antimicrobial [38,39] and anti-inflammatory [40] properties. Two of the main active components of OEO, which have been shown to provide beneficial effects on the intestinal health of pigs and on their productive performance, are carvacrol and thymol [35,41].

Knowing the chemical activities of these components, and therefore their instability, degradation due to the storage oxidizing environment of the feeds in silos, including exposure to oxygen, moisture and light is expected. In this way, allicin and carvacrol, major components identified in *Allium sativum* extract and *Origanum* essential oil, respectively, are known to be volatile and unstable compounds that, according to Liu et al. [42], in the presence of air and water are susceptible to degradation into diallyl disulfides (allicin oxidation) and thymoquinone (from the carvacrol oxidation) [43]. This situation justified the need to undertake a study regarding the stability of these feed additives, in both pre-starter and starter feeds, after their storage in silos.

In light of this, and as has been stated above, the inclusion of *Allium sativum* L. and *Origanum* essential oil and their combination in post-weaning piglet feed has been previously accomplished by different researchers [44]. But, according to the scientific literature, no research involving the stability of these bioactive components in the pre-starter and starter feeds under silo storage conditions has been accomplished before.

Thus, the main goals of the present research were to study the effect of feeding different concentrations of OEO and PGP on intestinal histological parameters in the jejunum and ileum on weaned piglets; to analyze the impact of these compounds on intestinal microbiota indicators (*E. coli* and *Lactobacillus* spp.); to monitor AMR in the commensal indicator *E. coli*; and to evaluate the degradation of the bioactive components of OEO and PGP after their storage in silos.

## 2. Materials and Methods

### 2.1. Additives and Feed Composition

Data on the composition of the control diet, the bioactive components of OEO, and the chemical and amino acid composition of PGP are described in Rivera-Gomis et al. [44]; the additives and feed composition are described in Serrano-Jara et al. [45].

Following weaning, for two weeks, the piglets received a pre-starter feed. During the following weeks, they were given a starter food, until they were ten weeks old.

The control diet was formulated to satisfy the nutritional and energy requirements of piglets during the post-weaning period; the main OEO compounds were carvacrol (70.32%) and thymol (4.10%) encapsulated by a coating of mono- and diglycerides of edible fatty acids and hydrogenated sunflower fat in a size of 800 μm. The PGP contained 63% of purple garlic in the form of mashed and dried powder with silicic acid (E-551) and citric acid as additives.

The additives and feed composition are described in Serrano-Jara et al. [45].

### 2.2. Animals, Housing and Experimental Design

The University of Murcia, through the Ethical Committee for Animal Experimentation (CEEA), approved the experimental protocols used in the study (Authorization Code 471/2018). Animal handling was carried out in accordance with current legislation on animal welfare in the EU.

The animal population studied was located in the facilities of the company Dalland Hybrid España S.A (DHSA, Murcia, Spain). The piglets were crosses of Pietrain, Large White and Landrace. Weaning was carried out at twenty-one days of age, and slaughter at ten weeks of age. The animals were housed in commercial farm conditions.

For the development of this research, seven experimental treatments were manufactured and distributed among seven groups of piglets. A basal diet (control group) without ZnO or experimental additives added; ZnO (2500 mg of Zn/kg of feed) (2); OEO 0.4% and 1.2% PGP 0.4% and 2% and a mixture of OEO 1.2% and PGP 2%. ZnO was administered as Zincotrax (Andrés Pintaluba S.A., Tarragona, Spain).

A total of 3000 animals participated in the experiment [46]. For sampling, 140 animals divided into 10 replicates were used. The experimental unit was the replicate. Each replicate had the seven experimental groups described above. The number of animals included in each replicate was 14, and within each replicate, 2 animals were assigned to each experimental group and sampled. Each replicate lasted 7 weeks, from weaning to the end of the transition period (Table 1).

### 2.3. Quantitative Analysis of Bioactive Components in the Feed: Storage Stability

Two batches of feed, pre-starter and starter enriched with the additives under study were manufactured right before their inclusion in the animal diet. During the storage period, at the commercial farm, samples from the silos were taken at the beginning, and at the end of every experimental diet, corresponding the latter with the transition stage to fattening and, therefore, with the end of the experimental trials. In this way, the pre-starter and starter were kept in silos for 15 and 34 days, respectively. All samples were placed in vacuum bags and stored at −80 °C until the time of analysis.

For the extraction and analysis of the bioactive components in both feeds, different protocols attending to the chemical nature of every additive extract were developed. All the samples were analysed in triplicate.

#### 2.3.1. Extraction of Bioactive Compounds from Feed Enriched with PGP

In the case of PGP, samples taken from the silo were homogenised and ground to a particle size < 0.5 mm. For the isolation of the bioactive components, a solid/liquid extraction method was undertaken. An amount of 2 mL of extracting agent (70% MeOH, 10% H_2_O, 10% acetone and 10% chloroform), enriched with 5 ppm of hesperidin as internal standard, was added to 250 mg of ground material. The mixture was shaken at 900 oscillations/min (Vibromatic, Selecta, Spain) for 30 min, and centrifuged for 10 min at 1480× *g* to separate the solid residue from the extract.

#### 2.3.2. HPLC–ESI-MS/TOF Analysis of PGP Feed Extracts

The chemical characterization of the PGP extracts was performed using Liquid Chromatography (Agilent Technologies, Santa Clara, CA, USA, 1200 series) coupled with High-resolution Mass Spectrometry (LC–QTOF MS/MS), using a hybrid Quadruple Time-of-Flight Detector (Agilent Technologies, model 6540) as it was previously described by Molina-Calle et al. [46], with some modifications. For the chromatographic separation, a reverse phase C18 column (Zorbax Eclipse Plus C18 (Santa Clara, CA, USA) Rapid Resolution HD 3.0 × 150 mm, 1.8 µm) was used. The mobile phase was H_2_O (A) and acetonitrile (B), both eluents acidified at 0.1% (*v*/*v*) with formic acid. The elution gradients were as follows: 0 to 1 min, 4% (B); 1 to 6 min, increase from 4% to 40% (B); from 6 to 10 min, increase from 40% to 100% of (B); and, finally, for 10 to 20 min, to ensure elution of all sample components, it was maintained in 100% (B). The flow rate was 0.25 mL/min and 2 µL of sample were injected. The mass spectrometry analysis was carried out using an electrospray ionization source, operating in a negative ionization mode. The detection was performed considering a mass range of 60–1200 *m*/*z*, using different collision energies (20 and 40 eV). For the quantification of alliin and γ-glutamyl-S-allylcysteine, linear regression models were applied using standard dilution techniques in a quantification range from 0.1 to 10 mg/mL. 

#### 2.3.3. Extraction of Bioactive Compounds from Feed Enriched with OEO

As has been described in Section 2.3.1, homogenised samples of feeds were ground to a particle size < 0.5 mm, and a solid/liquid extraction method was performed. In this case, 1 g of pulverized feed was extracted with 40 mL of a mixture of organic solvents (hexane/ethyl acetate 60/40 *v*/*v*) by sonication at 40 °C for 15 min, followed by stirring for 30 min with a magnetic stirrer at the same temperature and inert atmosphere. The resulting mixtures were centrifuged for 10 min at 2000× *g* and the supernatants were taken to dryness at 40 °C under vacuum conditions in an evaporator system (Syncore Polyvap R-96) (Büchi, Flawil, Switzerland). The dried residues were redissolved in methanol and made up 2 mL. The extracts were kept in vials at −80 °C until their corresponding analysis.

#### 2.3.4. HPLC Analysis of OEO Feed Extracts

The qualitative and quantitative analysis of the major components of OEO (the phenolic components carvacrol and thymol) were carried out using High-pressure Liquid Chromatography coupled to a Diode Array Detector device (HPLC-DAD 1200 Series, Agilent, Waldbronn, Germany). The chromatographic separation was performed on a reverse-phase ZORBAX SB-C18 column (4.6 × 250 mm, 5 µm pore size, Agilent Technologies, USA) using a guard column (ZORBAX SB-C18 4.6 × 125 mm, 5 µm pore size, Agilent Technologies, USA), at ambient temperature. The mobile phase was acidified H_2_O (0.05% formic acid) (A) and acetonitrile (B). The gradient used was as follows: 0 min, 50% B; 5 min, 52% B; 10 min, 55% B; 13 min, 90% B; 15 min, 100% B (held until min 22); 25 min, 50% B, with a flow rate of 1 mL/min. The wavelength of detection was set at 210 nm. Before injection, samples were passed through a 0.45 μm filter (Millipore SAS, Molsheim, France) and 20 μL were injected. For the quantification of carvacrol and thymol linear regression models were calculated using standard dilution techniques. Results were expressed as mg of phenolic compound/g of feed.

### 2.4. Sample Collection and Preparation

#### 2.4.1. Samples for the Study of the Histological Parameters

The samples were taken at the end of the experimental period when the piglets were ten weeks old. The number of animals sampled was 2 per experimental group in each replicate, 140 in total. All the animals sacrificed to obtain samples weighed 20 ± 1 kg. The samples were obtained from the jejunum and ileum. Samples were taken from each anatomical region: 140 from the jejunum and 140 from the ileum.

Sample processing was carried out as in Serrano-Jara et al. [45]. The samples were stained with Hematoxylin–Eosin (HE). Subsequently, morphometric evaluation was carried out with an optical microscope at 10× magnification. In each sample, 10 villi were located. The choice of villi was based on the location of the quiliferous duct. The height and width of the villi and the depth of the crypt were measured using the SPOT Advanced software version 4.0.5 (SPOT Imaging, Diagnostic Instruments Inc., Sterling Heights, MI, USA).

Villus height was measured from the final tip of the villus to the base, where the crypt begins; the width of the villus was measured by drawing a perpendicular line from one lateral end of the villus to the other; the depth of the crypt was made from its attachment to the villus to the beginning of the muscular layer of the mucosa. Subsequently, the villus height/crypt depth index was calculated.

#### 2.4.2. Samples for the Study of Intestinal Microbiome Indicator Bacteria

To assess the intestinal flora of the piglets, the isolation and identification of gut health indicator bacteria (*E. coli* and *Lactobacillus* spp.) was carried out.

Serial decimal dilutions (−1, −2, −3, −4, −5, −6, −7, −8, −9) were made from 140 cecal content samples (±1 g) to detect and quantify the presence of commensal *E. coli* and *Lactobacillus* spp. isolation was performed by culturing the different dilutions in the specific media for the aforementioned microorganisms: *E. coli* in RAPID’*E. coli* and *Lactobacillus* spp. in Rogosa.

#### 2.4.3. Study of *E. coli* AMR

Due to time and budget constraints, the AMR study was only performed in samples from the following experimental groups that included the control, ZnO and the higher doses of additives studied: OEO 1.2%, PGP 2% and OEO 1.2% + PGP 2%.

Seven antibiotics were used: ciprofloxacin (fluoriquinolones), nalidixic acid (fluoriquinolones), ceftazimide (cephalosporins), colistin (polymyxin), ampicillin (beta lactams), gentamicin (amino glycosides) and tetracycline (tetracyclynes).

The Minimum Inhibitory Concentration (MIC) of each antibiotic was used to monitor resistance in the commensal indicator *E. coli* taken from cecal content samples. The procedure consisted of determining the growth of the microorganism in the presence of increasing concentrations of the antimicrobial, which was diluted in the culture medium. Its MIC was considered at the lowest concentration of antibiotic at which there was no growth.

The resistance profile to antibiotics for veterinary use was analysed in 81 randomly selected *E. coli* isolates, distributed among the different study groups included in this part of the experiment. All the isolates were analysed at the end of the study to have the same analytical conditions during the laboratory work. The *E. coli* strain (CECT 434) was used as a control microorganism, which is the strain recommended by the Spanish collection of type cultures for MIC tests in clinical isolates.

Antibiotics were prepared from their commercial standards and diluted in a stock dilution, which was frozen in aliquots and kept at −80 °C. At the time of use, an aliquot was thawed and serial solutions were made in the cut-off ranges and concentration intervals indicated in Decision 2013/652/UE [47].

A Trypticasein Soy Broth (TSB) suspension for bacterial growth was prepared from a 24 h culture and compared to a turbidity of 0.5 Mc Farland. In 96-well plates, the micro-dilution of the isolate and the antibiotics was carried out, obtaining the appropriate concentration. All the standardizations of the bacteria were confirmed by seeding and quantification in a selective medium.

To perform the MIC tests, the concentration intervals (mg/L) and the epidemiological cut-off value specified in Decision 2013/652/UE [47] were used. The MICs at which 50% and 90% of the isolates were inhibited by the antibiotic were defined as MIC50 and MIC90 respectively.

The categories established by the European Medicines Agency were used to classify the antibiotics for which the resistance of the *E. coli* isolate was evaluated.

### 2.5. Statistical Analysis

The statistical variables of the OEO and the PGP components were carvacrol and thymol and γ-glutamyl-S-allylthio-cysteine, γ-glutamyl-S-methylcysteine, γ-glutamyl-S-allycysteine, total sulphur compounds and aliin. The statistical variables of intestinal morphometry studied were jejunum and ileum villus height, thickness and depth. Colony counts of commensal *E. coli* and *Lactobacillus* spp. were used as statistical variables. The values for intestinal morphometry parameters and colony counts of the two samples per replicate were averaged. Proportions and frequency values were used to quantify AMR values in *E. coli*.

The data were analysed using the statistical software IBM SPSS Statistics (version 26.0). All data were evaluated for normality using the Shapiro–Wilk test. Data were normally distributed and presented as mean  ±  standard deviation of the mean (SD) and compared using the one-way ANOVA test, followed by Tukey*’*s multiple comparisons test. The value of *p*  <  0.05 was used to indicate significance in all analyses.

## 3. Results

### 3.1. Quantification of Major Active Components of Oregano Essential Oil in Pre-starter and Starter Feeds

The results concerning the variation in concentration of carvacrol and thymol in both feeds, from the beginning to the end of the experimental stage are shown in Table 2.

A statistically significant (*p* < 0.05) detriment in the concentration of these two bioactive components was detected. A loss close to 30% in the starter feed supplemented with 1.2% of OEO was quantified after 34 days of storage.

### 3.2. Analysis of Bioactive Components of PGP in the Feed Preparation

Related to the analysis of bioactive components of PGP in the feed preparation, the chromatographic analysis (HPLC-MS/MS) allowed for the identification of a total of nine major sulphur components, mainly derived from cysteine. Among them, alliin and γ-Glutamyl-S-allylcysteine (representing almost two thirds of the total content of sulphur compounds identified) stand out as the major compounds quantified (Table 3).

The total content refers to the sum of all sulphur compounds, including γ-l-glutamyl-S-(2-carboxy-1-propyl) cysteinylglycine, allyl ethyl sulphide, S-1-propenyl-l-cysteine, S-allylcistein and S-propyl-l-cistein, that were quantified as γ-glutamyl-S-allyl-cysteine equivalents.

The organosulphur profile of the PGP added to the feeds was analysed at the end of both feeding stages (pre-starter and starter). In the pre-starter feed, for the three enriched diets, the reduction in concentration after 15 days of storage ranged from 9 to 13%. However, after 34 days, the degradation process occurred to a bigger extent, and the range of losses varied between 30 and 36% with respect to the initial concentration. 

### 3.3. Intestinal Morphometry Parameters

Regarding the height of the villi, no significant differences were found between the treatments (*p* > 0.05) (Table 4).

The results referring to the width of the villi did show significant differences (*p* < 0.05). The highest values corresponded to the PGP 0.4% (162.15 ± 14.06 µm) and the control group (160.11 ± 17.33 µm), which showed significant differences with the OEO 1.2% (141.80 ± 17.43 µm) and the combined dose of OEO 1.2% and PGP 2% (130.86 ± 11.88 µm) that obtained the lowest values (Table 4).

Regarding the depth of the crypts and the relationship between villi height and crypt depth, no significant differences were found between the treatments (*p* > 0.05) (Table 4).

In relation to the height of the villi, no significant differences were found between the treatments (*p* > 0.05) (Table 5).

The results for the width of the villi did present significant differences (*p* < 0.05). The highest value corresponded to PGP 0.4% (170.50 ± 22.19 µm), which showed significant differences with the combined dose of OEO 1.2% and PGP 2% (140.43 ± 11.45 µm), and OEO 1.2% (145.49 ± 18.39 µm) and OEO 0.4% (153.11 ± 13.82 µm), but not with the other treatments (Table 5). 

Regarding the depth of the crypts and the relationship between the height of the villi and the depth of the crypts, no significant differences were found between the treatments (*p* > 0.05) (Table 5).

### 3.4. Quantification of Intestinal Microbiota Indicators from Cecal Content

In relation to the effect on the *E. coli* counts of the high and low doses of the additives studied (Table 6), there were only significant differences between the control group and the ZnO (*p* < 0.05).

Regarding the counts of *Lactobacillus* spp. and the *Lactobacillus*: *E. coli* ratio (L:E) (Table 6), statistically significant differences were not observed.

### 3.5. AMR Monitoring in Commensal E. coli

The values of MIC50 and MIC90 (Table 7), all values were above the epidemiological cut-off value (>16). In relation to the resistance percentages found (Table 8), with respect to category A, there was 100% resistance to nalidixic acid in all the groups. Regarding ciprofloxacin, values above 50% resistance were observed in all groups. 

For category B (Table 7 and Table 8), the lowest percentages of resistance in the study were found. Regarding ceftazidime, the lowest resistance values were found in the control group (17%) and the highest in OEO 1.2% (73%) and PGP 2% (50%). The MIC50 was below the epidemiological cut-off value (>0.5) for all groups, except for the OEO 1.2%, which had a MIC50 of 1.

Regarding colistin, there was 0% resistance in the OEO 1.2%. The highest percentages of resistance were found in ZnO (25%) and PGP 2% (25%). In relation to the MIC50, all groups had values below the epidemiological cut-off value (>2). The lowest MIC90 value was for the OEO 1.2% group (≤1).

For category C (Table 7 and Table 8), ampicillin and gentamicin were studied. For ampicillin, there were very high percentages of resistance, reaching 100% for PGP 2% and OEO 1.2%. MIC50 and MIC90 values were above the maximum concentration studied (>64).

In relation to gentamicin, 100% of the isolates studied in the PGP 2% group were resistant. The resistance values were above 80% for all groups. All MIC50 and MIC90 values were above the *E. coli* epidemiological cut-off value for gentamicin (>2).

The only category D (Table 7 and Table 8), antibiotic studied was tetracycline. The lowest percentage of resistance was that of the OEO 1.2% group (36%). The highest value was shown by the PGP 2% (92%).

Regarding the MIC50 values, the group that showed values below the epidemiological cut-off value (>8) was the OEO 1.2% (≤2). All the groups studied had a value for MIC90 of >64 except ZnO, which had a value of 64.

## 4. Discussion

One of the major concerns regarding the bioactivity of natural matrices is their processing and storage conditions. Deviations in relative concentration or even degradation of these active components may lead to deep variations in their efficacy of use [48].

Previous assays, regarding the stability of the encapsulated OEO stored for three months at room temperature (25 °C), were accomplished in our laboratory. Results showed high stability of the carvacrol content in the charged micro-particles since the concentration varied from 73 ± 4.24 to 70 ± 2.26 (mg of carvacrol/g encapsulated particle) at the end of the experiment (unpublished results). In this research, under unfriendly environmental conditions, the losses observed in the OEO phenolic components could be associated with the relatively high temperatures in this Mediterranean area, along with the possible oxidation processes, and their potential chemical interactions with other components of the feed matrix [49].

In line with this, Güneş et al. [50] published that the oxidation of these phenolic components may result in the formation of thymo-hydroquinone and benzoquinones. However, later studies carried out by Soliman et al. [51] pointed to the stability of carvacrol and thymol under oxidative conditions (hydrogen peroxide-induce degradation), and after photo and thermal degradation studies. This agrees with our findings since the HPLC analysis of the feeds after storage did not reflect the presence of these quinone degradation products. In relation to the chemical interactions of carvacrol with some constituents of the feed matrices, Wang et al. [49] showed the effect of the food composition on carvacrol migration. For these authors, the fat content has significant effects on carvacrol absorption in ground beef products, reducing at a significant level the antimicrobial activity of this phenolic component. Relative to the third factor under study, temperature, a decrease near 61.84% in carvacrol content was described by Fraj et al. [52] in polycaprolactone nanocapsules/microspheres of OEO stored at 40 °C.

According to these results, it could be considered that the losses of phenolic compounds from OEO in both feeds are mostly associated with the temperature (21 ± 10 °C) of the local area (Spanish Southeast) during the experimental trial (April–December, 2019) and their possible interactions with some components of the feed matrices.

The presence of organosulphur compounds in garlic has been previously described by several authors in the scientific literature. Thus, Salehi et al. [53] described allicin as the main bioactive compound in garlic representing 70–80% of the total organosulphur content. However, for Moreno-Ortega et al. [54] in fresh garlic, cycloalliin was the major organosulphur component identified. A different profile to the one described for PGP in Table 3 was found in whole garlic by Amagase et al. [55]. For these authors, the organosulphur profile of intact garlic is typically composed of 1% alliin, together with (1)-S-methyl-l-cysteine sulfoxide (methiin) and (1)-S-(trans-1-propenyl)-l-cysteinesulfoxide, S-(2-carboxypropyl) glutathione, γ-glutamyl-S-allyl-l-cysteine, γ-glutamyl-S-(trans-1-propenyl)-l-cysteine and γ-glutamyl-S-allyl-mercapto-l-cysteine.

The purple garlic used in this study is a native ecotype of *Allium sativum*, so differences in quantitative composition can be attributed to the intraspecific variability that normally occurs among ecotypes of this species [56].

One of the major concerns regarding the use of these organosulphur extracts is their lack of chemical stability. It is well known that these active compounds are susceptible to degradation, volatilization and oxidation when exposed to harsh environmental conditions, such as high temperature, oxygen, and light [57].

The losses in the organosulphur profile were expected since, according to Horníčková et al. [58], one of the main groups of components that increase their concentrations, because of a degradation process in garlic, are S-alk(en)ylcysteine sulfoxides. These components come from the conversion of the corresponding c-glutamyl dipeptides to sulfoxides. The study of the degradation process was not included among the main objectives of this work, but rather it was intended to guarantee that at the end of the experimental trial, the animals were receiving an adequate concentration of bioactive components that help them improve their intestinal health.

This study was carried out on a commercial farm where the frequent use of vegetable additives makes it essential to study the degradation of their active components and their effects.

Regarding the morphometry of the villi in our study, the highest doses of PGP and OEO and their combination obtained the most favorable results, surpassing in some cases the values for the ZnO group. The group that received the combination of OEO and PGP showed longer and narrower villi and less deep crypts in the jejunum and ileum. This effect has a direct impact on intestinal absorption and the consequent growth since a greater surface area implies greater nutrient absorption [59,60]. Tatara et al. [61] published positive results based on the use of aged garlic extract and allicin on the morphometric properties of broilers' intestinal villi. OEO and PGP in high doses and combined have been found to have a positive effect on the intestine, both structurally and immunologically [47]. However, low doses at 0.4% had a better effect on oxidative stress parameters [42]. The results indicate the need to continue refining the correct dosage of these additives to reach their full potential.

In our research, we see how the highest count of *Lactobacillus* spp. colonies appear to be using the PGP 2%. But, there are no significant differences between the different groups. Regarding the *E. coli* count, the ZnO group did not show significant differences with any of the additives used. Among the additives, the high doses and their combination showed the lowest *E. coli* values.

Likewise, the fecal count of *E. coli* was significantly reduced in a study on the supplementation of this additive in piglets [61]. Also, it was observed in a study carried out on adult sows that when supplementing with garlic powder, there was less proliferation of pathogens in the intestinal microbiota. This decrease or absence of pathogenic species in the microbiota of cattle means that its use can be considered in the future as an effective alternative in the prevention of some bacterial diseases. In addition, garlic can enhance the cellular and humoral response of the immune system by stimulating various cells or mechanisms [62]. It should be noted that various scientific investigations show that garlic extract improves the digestibility of nutrients in broilers and increases feed intake in lactating pigs.

Regarding the AMR monitoring in commensal *E. coli* from cecal content, the isolates from the OEO 1.2% group showed no resistance to colistin and presented the lowest resistance values for most of the studied antibiotics in comparison with the rest of the experimental groups.

In other studies, on OEO, it was shown how carvacrol inhibited flagellin, and, therefore, flagellar development, leaving *E. coli* O157:H7 immobile due to a heat shock of protein [63]. In addition, numerous in vitro studies show that this additive has a lower Minimum Inhibitory Concentration (MIC) for pathogenic species such as *E. coli*, *Clostridium* spp. and *Salmonella* spp., in relation to commensal or positive as *Enterococcus* spp. or *Lactobacillus* spp. That is, it can favor the growth of beneficial flora, and, therefore, the intestinal health of animals [64].

We observed high levels of AMR in antimicrobials from all categories. This is a worrisome result, even more so considering that antimicrobials from categories that are not used in animal production were also studied. However, we did not identify any specific patterns of resistance due to sampling and testing limitations. Therefore, further investigation should be carried out to identify AMR patterns in pigs and possible relations with the use of feed additives.

## 5. Conclusions

Despite the degradation in the absolute concentration of the phenolic and allylic components in the feeding trial, the remaining concentration was still active, which could be related to the beneficial effect of the components used.

OEO 1.2%, PGP 2% and their combination showed a similar or superior beneficial effect to ZnO on the studied parameters of intestinal villi and crypts. The additives, especially high doses, positively influenced the regulation of the intestinal microbiota of piglets during the transition phase, they showed similar and lower levels than ZnO in the *E. coli* count and similar or higher in that of *Lactobacillus* spp. Both nutraceuticals show potential as possible substitutes for ZnO and further study is needed to better define their effects and establish their dosage.

The *E. coli* isolates studied showed very high resistance levels to antimicrobials from several categories, including some reserved for human medicine. This finding highlights that proper regulation of the use of antimicrobials is of vital importance to limit the spread and prevent the development of AMR.

## Figures and Tables

**Table 1:** Distribution of groups, replicates and animals.

Group	Replicates	Animal/Replicate	Animal/Group
Control Grup	10	2	20
ZnO	10	2	20
OEO 0.4%	10	2	20
OEO 1.2%	10	2	20
PGP 0.4%	10	2	20
PGP 2%	10	2	20
OEO 1.2% + PGP 2%	10	2	20
Total		14	140

OEO: oregano essential oil; PGP: purple garlic powder.

**Table 2:** Quantification of major active components of oregano essential oil in pre-starter and starter feeds.

	OEO %	Storage Days	Carvacrol (mg/kg Feed)	Thymol (mg/kg Feed)
Pre-starter	0.4%	0	275.32 ± 18.99 ^a^	14.73 ± 0.98 ^a^
		15	244.74 ± 2.07 ^b^	13.14 ± 0.14 ^b^
	1.2%	0	856.31 ± 27.44 ^a^	46.67 ± 1.23 ^a^
		15	720.09 ± 1.95 ^b^	39.32 ± 1.36 ^b^
	1.2% + PGP 2%	0	930.52 ± 30.81 ^a^	49.44 ± 1.55 ^a^
		15	794.33 ± 38.54 ^b^	46.13 ± 1.79 ^b^
Starter	0.4%	0	214.92 ± 6.35 ^a^	13.10 ± 0.30 ^a^
		34	187.54 ± 3.84 ^b^	11.44 ± 0.40 ^b^
	1.2%	0	845.72 ± 33.5 ^a^	46.34 ± 1.45 ^a^
		34	583.38 ± 9.74 ^b^	30.16 ± 1.34 ^b^
	1.2% + PGP 2%	0	917.89 ± 42.23 ^a^	44.74 ± 2.73 ^a^
		34	655.63 ± 11.23 ^b^	32.92 ± 2.18 ^b^

The letters (a, b) indicate statistically significant differences between the days of storage (considered within each level of supplementation and its corresponding feed) at *p* ≤ 0.05. Values are (*n* = 3 ± standard deviation); OEO: oregano essential oil; PGP: purple garlic powder.

**Table 3:** Quantification of major active components of purple garlic powder in pre-starter and starter feeds.

	D	γ-Glutamyl-S-Allylthio-Cysteine	γ-Glutamyl-S-Methylcysteine	γ-Glutamyl-S-Allylcysteine	Total Sulphur Compounds (mg/kg)	Aliin
PGP 0.4%						
Pre-starter	0	18.72 ± 1.51 ^a^	18.82 ± 1.33 ^a^	101.79 ± 1.70 ^a^	338.72 ± 9.16 ^a^	5.13 ± 1.06
	15	17.06 ± 1.39 ^a^	17.12 ± 1.10 ^a^	91.61 ± 4.03 ^b^	314.07 ± 11.42 ^b^	4.88 ± 0.92
Starter	0	17.63 ± 2.54 ^a^	19.48 ± 1.47 ^a^	113.82 ± 15.74 ^a^	343.81 ± 7.98 ^a^	5.57 ± 0.63
	34	12.24 ± 1.84 ^b^	13.24 ± 0.98 ^b^	80.46 ± 3.88 ^b^	271.17 ± 16.04 ^b^	4.67 ± 0.64
PGP 2%						
Pre-starter	0	304.92 ± 10.74 ^a^	193.14 ± 5.83 ^a^	1309.30 ± 27.74 ^a^	1913.27 ± 30.52 ^a^	141.6 ± 6.46 ^a^
	15	270.07 ± 7.72 ^b^	171.95 ± 3.92 ^b^	1169.53 ± 35.14 ^b^	1692.44 ± 15.55 ^b^	132.9 ± 6.42 ^a^
Starter	0	311.12 ± 7.26 ^a^	187.74 ± 4.63 ^a^	1275.35 ± 35.93 ^a^	1896.86 ± 42.15 ^a^	146.5 ± 8.45 ^a^
	34	215.23 ± 5.51 ^b^	119.67 ± 6.81 ^b^	844.61 ± 1.71 ^b^	1312.71 ± 54.85 ^b^	119.7 ± 8.51 ^b^
PGP 2% + OEO 1.2%						
Pre-starter	0	296.49 ± 6.66 ^a^	190.18 ± 2.60 ^a^	1279.78 ± 36.28 ^a^	1872.91 ± 72.16 ^a^	136.75 ± 4.14 ^a^
	15	264.30 ± 4.18 ^b^	170.08 ±7.15 ^b^	1124.33 ± 32.75 ^b^	1637.55 ± 26.70 ^b^	126.71 ± 8.25 ^a^
Starter	0	306.38 ± 10.73 ^a^	186.65 ± 7.20 ^a^	1278.07 ± 42.71 ^a^	1918.79 ± 68.07 ^a^	141.35 ± 8.06 ^a^
	34	217.72 ± 13.83 ^b^	124.33 ±3.02 ^b^	855.41 ± 7.34 ^b^	1382.76 ± 96.07 ^b^	116.53 ± 9.83 ^b^

The letters (a, b) indicate statistically significant differences between the days of storage (considered within each level of supplementation and its corresponding feed) at *p* ≤ 0.05; values are (*n* = 3 ± standard deviation); OEO: oregano essential oil; PGP: purple garlic powder.

**Table 4:** Intestinal morphometry parameters (µm) of the jejunum for the different experimental groups (n = 20).

Group	JVH	JVT	JCD	JVH:JCD
	Mean ± SD	Mean ± SD	Mean ± SD	Mean ± SD
Control Group	406.39 ± 57.16	160.11 ± 17.33 ^c^	287.53 ± 46.84	1.46 ± 0.40
ZnO	387.36 ± 66.58	147.81 ± 12.78 ^bc^	279.50 ± 44.42	1.43 ± 0.37
OEO 0.4%	402.00 ± 53.79	154.50 ± 16.16 ^bc^	298.73 ± 56.33	1.40 ± 0.33
OEO 1.2%	421.84 ± 50.80	141.80 ± 17.83 ^ab^	282.07 ± 50.40	1.54 ± 0.32
PGP 0.4%	380.02 ± 58.56	162.15 ± 14.06 ^c^	272.15 ± 46.57	1.46 ± 0.44
PGP 2%	414.84 ± 59.12	152.15 ± 16.00 ^bc^	268.01 ± 34.08	1.58 ± 0.35
OEO 1.2% + PGP 2%	436.43 ± 49.18	130.86 ± 11.88 ^a^	261.12 ± 35.64	1.70 ± 0.25
*p*-value	>0.05	<0.05	>0.05	>0.05

The letters (a, b, c) indicate statistically significant differences between the groups (*p* ≤ 0.05); JVH: jejunum villus height; JVT: jejunum villus thickness; JCD: jejunum villus depth; SD: standard deviation. OEO: oregano essential oil; PGP: purple garlic powder.

**Table 5:** Intestinal morphometry parameters (µm) of the ileum for the different experimental groups (*n* = 20).

Group	IVH	IVT	ICD	IVH:ICD
	Mean ± SD	Mean ± SD	Mean ± SD	Mean ± SD
Control Group	366.96 ± 65.93	154.81 ± 13.79 ^ab^	294.98 ± 43.48	1.28 ± 0.32
ZnO	405.78 ± 62.01	155.60 ± 11.15 ^ab^	285.86 ± 51.56	1.47 ± 0.35
OEO 0.4%	376.48 ± 67.02	153.11 ± 13.82 ^a^	288.86 ± 35.11	1.32 ± 0.26
OEO 1.2%	395.61 ± 50.70	145.49 ± 18.39 ^a^	270.41 ± 50.11	1.53 ± 0.43
PGP 0.4%	369.33 ± 33.87	170.50 ± 22.19 ^b^	282.43 ± 41.49	1.34 ± 0.24
PGP 2%	387.17 ± 49.77	156.31 ± 17.51 ^ab^	266.84 ± 31.68	1.48 ± 0.31
OEO 1.2% + PGP 2%	416.07 ± 31.08	140.43 ± 11.45 ^a^	268.79 ± 34.15	1.58 ± 0.28
*p*-value	>0.05	<0.05	>0.05	>0.05

The letters (a, b) indicate statistically significant differences between the groups (*p* ≤ 0.05); IVH: ileum villus height; IVT: ileum villus thickness; ICD: ileum villus depth; SD: standard deviation. OEO: oregano essential oil; PGP: purple garlic powder.

**Table 6:** Values of the microbiological counts of *E. coli* and *Lactobacillus* spp. from cecal content (*n* = 20).

Group	*E. coli* ± SD	*Lactobacillus* spp. ± SD	L:E ± SD
Control	5.37 ± 1.56 ^a^	6.71 ± 0.88	1.35 ± 0.49
ZnO	6.56 ± 1.29 ^b^	6.88 ± 1.04	1.06 ± 0.21
OEO 0.4%	6.14 ± 1.06 ^ab^	7.09 ± 0.77	1.17 ± 0.17
OEO 1.2%	5.47 ± 0.96 ^ab^	7.16 ± 1.31	1.26 ± 0.17
PGP 0.4%	5.79 ± 1.17 ^ab^	6.62 ± 0.74	1.17 ± 0.21
PGP 2%	5.80 ± 0.79 ^ab^	7.32 ± 1.14	1.27 ± 0.16
OEO 1.2% + PGP 2%	5.64 ± 1.13 ^ab^	6.45 ± 0.57	1.16 ± 0.13
*p*-value	<0.05	>0.05	>0.05

The letters (a, b) indicate statistically significant differences between the groups (*p* ≤ 0.05); SD: standard deviation; L:E is the ratio between *Lactobacillus* spp. and *E. coli*; OEO: oregano essential oil; PGP: purple garlic powder.

**Table 7:** Minimum Inhibitory Concentration (MIC) 50 and MIC 90 results for the studied *E. coli* isolates.

Group	Isolates	Antibiotics	MIC50	MIC90	Epidemiological Cut-Off Value
Control	12	Ciprofloxacin	2	>8	>0.064
		Nalidixic acid	>128	>128	>16
		Ceftazidime	≤0.5	>8	>0.5
		Colistin	1	8	>2
		Ampicillin	>64	>64	>8
		Gentamicin	8	>32	>2
		Tetracycline	16	>64	>8
ZnO	12	Ciprofloxacin	0.06	2	>0.064
		Nalidixic acid	64	>128	>16
		Ceftazidime	≤0.5	>8	>0.5
		Colistin	2	>16	>2
		Ampicillin	>64	>64	>8
		Gentamicin	8	>32	>2
		Tetracycline	64	64	>8
OEO 1.2%	18	Ciprofloxacin	0.12	1	>0.064
		Nalidixic acid	64	>128	>16
		Ceftazidime	1	8	>0.5
		Colistin	≤1	≤1	>2
		Ampicillin	>64	>64	>8
		Gentamicin	4	4	>2
		Tetracycline	≤2	>64	>8
PGP 2%	21	Ciprofloxacin	2	>8	>0.064
		Nalidixic acid	>128	>128	>16
		Ceftazidime	0.5	>8	>0.5
		Colistin	1	>16	>2
		Ampicillin	>64	>64	>8
		Gentamicin	>32	>32	>2
		Tetracycline	64	>64	>8
OEO 1.2% + PGP 2%	18	Ciprofloxacin	4	>8	>0.064
		Nalidixic acid	>128	>128	>16
		Ceftazidime	≤5	>8	>0.5
		Colistin	≤1	2	>2
		Ampicillin	>64	>64	>8
		Gentamicin	4	>32	>2
		Tetracycline	64	>64	>8

The number of isolates studied for each antibiotic was 81.

**Table 8:** Percentage of strains resistant to each antibiotic studied according to EMA category [19].

Group	Isolates	Ciprofloxacin	Nalidixic Acid	Ceftazidime	Colistin	Ampicillin	Gentamicin	Tetracycline
		Fluoroquinolones	Fluoroquinolones	Cephalosporins	Polymyxins	Beta Lactams	Amino Glycosides	Tetracyclines
		A	A	B	B	C	C	D
		%	%	%	%	%	%	%
Control	12	83	100	17	17	75	92	75
ZnO	12	50	100	33	25	83	92	67
OEO 1.2%	18	55	100	73	0	100	83	33
PGP 2%	21	92	100	52	25	100	100	92
OEO 1.2% + PGP 2%	18	61	100	22	6	83	94	61

Antimicrobial family and EMA category (A, B, C, D) have been included below each studied antibiotic.

## Data Availability

The datasets used and analysed during the current study are available from the corresponding author on reasonable request.
